# Estudios de ecología de la atención médica: análisis comparado en dimensión histórica, 1928-2018

**DOI:** 10.18294/sc.2023.4549

**Published:** 2023-11-22

**Authors:** Hugo Spinelli, Andrés Trotta, Viviana Martinovich, Marcio Alazraqui

**Affiliations:** 1 Doctor en Salud Colectiva. Investigador, Instituto de Salud Colectiva, Universidad Nacional de Lanús, Buenos Aires, Argentina. hugospinelli09@gmail.com Universidad Nacional de Lanús Instituto de Salud Colectiva Universidad Nacional de Lanús Buenos Aires Argentina hugospinelli09@gmail.com; 2 Doctor en Salud Colectiva. Director, Maestría en Epidemiología, Gestión y Políticas de Salud, Instituto de Salud Colectiva, Universidad Nacional de Lanús, Buenos Aires, Argentina. doctortrotta@gmail.com Universidad Nacional de Lanús , Maestría en Epidemiología, Gestión y Políticas de Salud Instituto de Salud Colectiva Universidad Nacional de Lanús Buenos Aires Argentina doctortrotta@gmail.com; 3 Profesora adjunta, Instituto de Salud Colectiva, Universidad Nacional de Lanús, Buenos Aires, Argentina. vivianamartinovich@gmail.com Universidad Nacional de Lanús Instituto de Salud Colectiva Universidad Nacional de Lanús Buenos Aires Argentina vivianamartinovich@gmail.com; 4 Doctor en Salud Colectiva. Director, Instituto de Salud Colectiva, Universidad Nacional de Lanús, Buenos Aires, Argentina. malazraqui@yahoo.com Universidad Nacional de Lanús Instituto de Salud Colectiva Universidad Nacional de Lanús Buenos Aires Argentina malazraqui@yahoo.com

**Keywords:** Atención Médica, Sistemas de Información, Encuestas, Hospitales

## Abstract

En la definición de las políticas de salud y en la toma de decisiones por parte de la gestión suele primar una separación entre clínica, epidemiología y salud pública, situación naturalizada desde la mirada de los hospitales y ministerios, pero artificial en los territorios, donde los problemas no se estructuran siguiendo la fragmentación de saberes, sino que se expresan en la complejidad de los problemas de las personas y los conjuntos poblacionales. Desde esa concepción, este trabajo recopila y analiza los estudios de ecología de la atención médica, realizados entre 1928 y 2018, que retoman el estudio precursor *“The ecology of medical care”*, de White, Williams y Greenberg, para centrar la discusión en tres ejes: 1) las regularidades presentes en los estudios de ecología de la atención médica, independientemente del año y el país; 2) los sistemas de información en salud y las encuestas de salud; y 3) la hegemonía institucional del hospital en el campo de la salud.

## INTRODUCCIÓN

En 1961, el *New England Journal of Medicine* publicó el artículo “*The ecology of medical care*”, en el que Kerr White, Franklin Williams y Bernard Greenberg^1^ se formulan tres preguntas: ¿llegan eficazmente a los consumidores los nuevos conocimientos de la enorme inversión pública en investigación médica?; ¿resultan óptimas la cantidad, calidad y distribución de la atención médica?; y ¿quién es el responsable de investigar estas cuestiones y de proporcionar datos sobre los que puedan basarse juicios certeros y programas eficaces? Esas preguntas mantienen total vigencia en cualquier país. Los autores^1^ parten de la premisa de que se sabe poco sobre los motivos por los cuales las personas, al percibir algún trastorno de su bienestar, buscan ayuda y dónde la buscan. Además, reconocen que, en la aceptación y utilización de la atención médica, el proceso está bajo el control de las propias personas.

En esa investigación se analizaron las decisiones que tomaron las personas mayores de 16 años ante síntomas, padecimientos, enfermedades o lesiones que afectaban su bienestar, para lo cual utilizaron seis categorías: población adulta expuesta a riesgo (1.000 personas); adultos que declaran una o más enfermedades o lesiones al mes; adultos que consultan a un médico una o más veces al mes; pacientes adultos hospitalizados al mes; pacientes adultos enviados a otro médico al mes; paciente adulto enviado a un centro médico universitario al mes^1^. Las dos primeras categorías remiten a la población, la tercera a la búsqueda de atención y las tres últimas a la atención médica^1^. Los resultados finales demostraron que de cada 1.000 personas, 750 declararon una o más enfermedades o lesiones, 250 consultaron a un médico una o más veces, 5 personas fueron enviadas a otro médico, 9 personas fueron hospitalizadas, y solo 1 fue enviada a un centro médico universitario^1^. 

Algunos detalles metodológicos para resaltar son que la unidad de tiempo fue el mes, la unidad de análisis fue el accionar de la persona ante sus dolencias y la decisión de los médicos y no el diagnóstico de la enfermedad, contradiciendo así toda una cultura en las formas de registrar los eventos en los sistemas de información en salud. El trabajo excluyó los embarazos no complicados, y a los menores de 16 años, debido a que los autores consideraron que sus decisiones suelen ser tomadas por los padres^1^. 

El trabajo de White *et al*.^1^^)^ se basó en publicaciones de la década de 1950 y los primeros años de la década de 1960^2^^,^^3^^,^^4^^,^^5^, y utilizó dos fuentes de datos de países diferentes: por un lado, los datos de los informes del Committe on the Costs of Medical Care para una muestra representativa de la población blanca de EEUU entre 1928 y 1931 y, por otro, los datos de la encuesta *The Survey of Sickness* representativa de la población de Inglaterra y Gales entre 1946 y 1950. 

**Figura 1:**
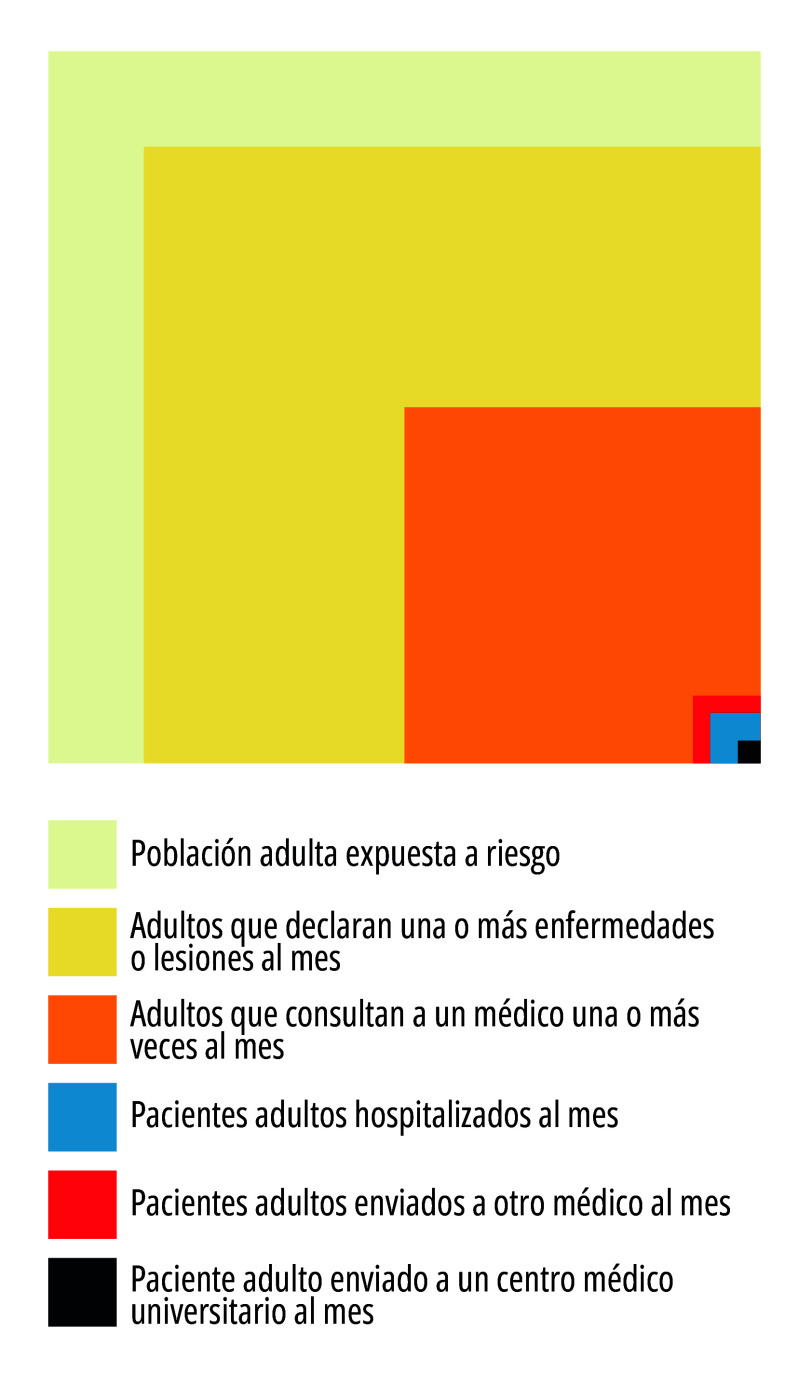
Estimaciones de la prevalencia mensual de enfermedad en la comunidad y de las funciones de los médicos, hospitales y centros médicos universitarios en la prestación de servicios médicos (adultos de 16 años y mayores). EEUU y Gran Bretaña (1928-1957).

Fuente: Elaboración propia a partir de White *et al*.^1^

En el estudio, White *et al*.^1^ señalan que solo una proporción de los malestares, padecimientos y síntomas que referencian las personas pasan a ser parte de la atención médica. Los resultados fueron graficados mediante una figura de cuadrados anidados (Figura 1), que denota la influencia de la forma en que graficaron sus hallazgos Horder y Horder^2^, quienes ya en 1954 habían descripto el patrón de enfermedades que recibía un médico general en Londres, trabajando con 2.000 consultas divididas en dos grupos de 1.000, correspondiente cada uno a los trimestres de verano y de invierno. Este estudio permitió conocer el perfil de padecimiento de las personas ante la práctica del médico generalista, para lo cual definió como unidad de análisis la consulta al médico por primera vez, y no la enfermedad. La definición del caso de estudio en la población resultó innovadora, por hacer foco en registrar la frecuencia relativa de determinadas condiciones mórbidas en la primera consulta, y por no usar el modo tradicional de medir la frecuencia relativa, registrando todas las consultas de un determinado padecimiento^2^. Este trabajo tuvo fuerte influencia en el trabajo de White *et al.*^1^. 

La época en que se publicó el artículo de White *et al*. se corresponde con lo que Starr llama “los años liberales de la salud” en EEUU, signado por el auge de una cultura hospitalocéntrica, que se expresa en el accionar del Congreso de ese país, que creó el programa federal conocido como Hill-Burton, que se propuso inaugurar 140.000 nuevas camas hospitalarias, lo que representaba un aumento del 40% del total de camas del país^6^^,^^7^^,^^8^. 

En 1996, 35 años después de la publicación de su artículo, White relata que al momento de publicarlo recibió numerosas cartas de colegas muy enojados por los contenidos del artículo, lo que lo llevó a pensar que el trabajo sería rápidamente olvidado^9^. En esa misma nota comenta que en 1973 volvió a revisar las conclusiones del trabajo publicado en 1961^1^ con datos del *National Center for Health Statistics* y los resultados se repetían. Además, destaca la importancia de recuperar al médico general, y lamenta que al momento de escribir el artículo desconociera los contenidos del *Dawson Report* del año 1920, realizado en Inglaterra, en el que se enuncia el concepto de atención médica primaria y se propone que la institucionalidad central sean los centros de salud primarios, cercanos a las comunidades, que tienen como referencia a los centros de salud secundarios (hospital)^10^. 

Respecto del uso del término ecología, White señala la resistencia que tuvo por parte del editor del *New England Journal of Medicine*, Joe Garland, para utilizar esa palabra en el título del artículo^9^. Pese a que White *et al*.^1^ no explicitan cabalmente el motivo de la utilización del término ecología, citaremos algunas referencias que nos aproximan a poder entender las influencias en las que podrían haberse basado.

La palabra ecología proviene del alemán Ökologie, término acuñado en 1866 por el zoólogo y biólogo Ernst H. Haeckel (1834-1919), a partir de las palabras griegas*oîkos*(casa) y*lógos*(tratado), haciendo referencia al estudio del lugar donde se vive. El concepto no fue muy utilizado hasta que, recién dos décadas y media después de su formulación, Ellen Swallow Richards (1842-1911) comienza a utilizarlo en EEUU. 

Ellen Swallow Richards fue la primera mujer en ingresar a una universidad de ciencias y al *Massachusetts Institute of Technology,* en EEUU, y tuvo un rol pionero en la relación entre la química y la alimentación^11^^,^^12^. Swallow es considerada una de las primeras feministas de EEUU y responsable de revolucionar la alimentación en la vida doméstica^13^. En 1870, Swallow junto a Mary Hinman Abel llevaron adelante una escuela para capacitar a las mujeres en situación de pobreza con el propósito de alimentar adecuadamente a sus familias^11^. Se la considera fundadora de la nutrición, de la economía del consumo, de la higiene ambiental y de la ciencia ecológica^14^^,^^15^^,^^16^. En 1911, año de su muerte, el *Journal of The American Public Health Association* publicó su obituario, lo cual señala su relevancia en el campo de la salud pública^17^*.*

Otra referencia sobre la ecología proviene de la Escuela de Sociología de Chicago, y de uno de sus primeros referentes, Robert Ezra Park (1864-1944), quien fue influenciado por John Dewey en *Harvard University*. Park es considerado uno de los principales referentes de dicha escuela junto a Thomas William^18^. Park defendió la idea de que el espacio físico era un espejo del espacio social, lo cual lo llevó a formular la noción de *ecología*, no en el sentido en que se usa en la actualidad, sino en el sentido que le da la biología, que la entiende como la competencia de la vida vegetal y animal por el espacio^19^^,^^20^^,^^21^. Entendemos que esta última concepción de la ecología podría ser la que toma White para su trabajo. 

El trabajo de White *et al*.^1^ fue replicado décadas después en distintos países. En este trabajo, nos proponemos hacer un análisis comparativo entre los resultados del artículo de White *et al*.^1^ y los estudios publicados de ecología de la atención médica que tomaron como objeto de estudio el país, para centrar la discusión en tres ejes: 1) las regularidades presentes en los estudios de ecología de la atención médica; 2) los sistemas de información en salud y las encuestas de salud; y 3) la hegemonía institucional del hospital en el campo de la salud. 

## METODOLOGÍA

Se realizó una búsqueda bibliográfica de los artículos científicos que siguieran el modelo utilizado por White *et al*. en “*The ecology of medical care*”^1^, para configurar un corpus bibliográfico de la literatura disponible. 

La búsqueda de artículos se realizó en las bases de datos Scopus, Pubmed, SciELO y Biblioteca Virtual de Salud. Se utilizaron las siguientes combinaciones de términos y operadores lógicos en cada una de las cuatro bases de datos bibliográficas mencionadas: a) “*ecology of medical care*” and “*health services*”; b) “*ecology of medical care*” and “*health services research*”; y c) “*ecology of medical care*” and “*health services utilization*”. 

La búsqueda bibliográfica se realizó en julio de 2023, y se incluyeron todos los artículos cuyo análisis se hubiera realizado a nivel país y que hubieran utilizado el modelo de cuadrados anidados de White *et al*.^1^^)^ como representación gráfica. No se incluyeron artículos cuyos resultados estuvieran a un nivel de agregación menor al de país -como provincia, municipio o equivalente- o en situaciones particulares de litigios territoriales como Taiwán y Hong Kong.

El corpus quedó conformado por nueve artículos, correspondientes a ocho países^22^^,^^23^^,^^24^^,^^25^^,^^26^^,^^27^^,^^28^^,^^29^^,^^30^. Tanto de Japón como de Corea del Sur se encontraron dos artículos de cada país. Para el análisis de los artículos seleccionados se agruparon los resultados en categorías compatibles con las utilizadas por White *et al*. en 1961^1^: a) Población estudiada; b) percibieron problemas de salud; c) buscaron atención; d) consultaron especialista/guardia; e) fueron hospitalizados; y f) fueron derivados a mayor complejidad. El análisis no incluyó la categoría “son derivados a otro médico”, utilizada por White *et al*., dado que no fue replicada en ninguno de los otros estudios. Además, para la categoría “buscan atención”, se estableció una subdivisión en dos subcategorías: “buscan atención: prácticas biomédicas” y “buscan atención: prácticas alternativas a la biomedicina”, dado que cuatro artículos analizados utilizaron esa división^22^^,^^23^^,^^26^^,^^28^ que consideramos relevante (Tabla 1).

**Tabla 1:** Número de personas en las categorías adaptadas de la propuesta de Kerr White, según países y períodos incluidos en este trabajo.

País	Período del estudio	Población estudiada	n	Perciben problemas de salud	n	Buscan atención				Consultan especialista/guardia	n	Son internados	n	Son derivados a mayor complejidad	n
						Prácticas alternativas	n	Prácticas biomédicas	n						
EEUU y Gran Bretaña^1^	1928-1957	Población de ≥16a	1.000	Declararon una o más enfermedades o lesiones al mes	750	-	-	Personas adultas que procuraron atención médica una o más veces al mes	250	Personas adultas derivadas a otra atención médica al mes	5	Personas adultas hospitalizadas al mes	9	Personas adultas enviadas a un centro médico universitario al mes	1
EEUU^22^	1996	Población general	1.000	Reportaron síntomas	800	Consultaron atención complementaria o alternativa	65	Consultaron atención médica en consultorio	217	Consultaron atención ambulatoria hospitalaria	21	Personas hospitalizadas	8	Persona hospitalizada en un centro médico académico	1
				Consideraron buscar atención médica	327			Recibieron atención médica domiciliaria	14						
								Consultaron atención médica en consultorio de atención primaria	113	Procuraron atención médica en emergencias	13				
Japón^23^	2003	Población general	1.000	Reportaron síntomas	862	Consultaron atención complementaria o alternativa	49	Consultaron atención médica en consultorio	307	Consultaron atención médica ambulatoria hospitalaria	88	Personas hospitalizadas	7	-	-
								Consultaron atención médica en atención primaria	232	Consultaron atención médica ambulatoria en hospital universitario	6				
								Recibieron atención médica domiciliaria	3	Consultaron atención médica en emergencias	10				
Japón^24^	2013	Población general	1.000	Reportaron síntomas	794	-	-	Consultaron atención médica en consultorio	265	Consultaron atención médica en centro médico universitario	10	Habían tenido una hospitalización	6	-	-
								Consultaron atención médica en atención primaria	206						
								Recibieron atención médica domiciliaria	7	Consultaron atención médica en emergencias	4				
Canadá^25^	2007	Población de ≥15a	1.000	Tenían una o más condiciones crónicas	561	-	-	Consultaron atención médica de medicina familiar	238	-	-	Pasaron la noche en un hospital	8	-	-
								Consultaron atención médica que no es medicina familiar	50						
								Consultaron atención de enfermería	32						
Austria^26^	2011	Población de ≥16a	1.000	Reportaron síntomas	646	-	-	Consultaron algún tipo de atención médica	460	Consultaron una especialidad en atención ambulatoria	206	Personas hospitalizadas	35	Personas hospitalizadas en un centro médico académico	3
				Consideraron atención médica	530			Consultaron atención de medicina general en consultorio	336	Consultaron una especialidad en atención ambulatoria hospitalaria	78				
Corea del Sur^27^	2012	Población de ≥18a	1.000	Tenían un problema de salud	939	Consultaron medicina oriental	38	Consultaron atención médica en una clínica	333	Consultaron atención médica ambulatoria en un hospital	101	Personas hospitalizadas en una clínica	3	Personas hospitalizadas en un hospital de tercer nivel	3
										Consultaron atención ambulatoria en un hospital de tercer nivel	35				
										Consultaron atención médica en emergencias	7	Personas hospitalizadas en un hospital	8		
Corea del Sur^28^	2018	Población de ≥19a	1.000	Tienen un problema de salud	763	-	-	Visitaron una clínica	344	Consultaron atención ambulatoria hospitalaria de segundo nivel	56	Consultaron atención ambulatoria en hospital de segundo nivel	4	Personas hospitalizadas en un hospital de tercer nivel	7
										Consultaron atención ambulatoria en hospital de tercer nivel	96				
										Consultaron atención médica en emergencias	9				
Israel^29^	2015-2016	Población de ≥15a	1.000	Reportaron síntomas	495	-	-	Recibieron asistencia médica	352	-	-	Personas hospitalizadas	15	-	-
				Consideraron buscar atención médica	450										
Suiza^30^	2018	Población de ≥18a	1.000	Habían tenido síntomas	546	Consultaron atención complementaria o alternativa	7	Consultaron asesoramiento médico	243	Consultaron un especialista	81	Recibieron atención en internación	21	Requirieron atención en unidad de cuidados intensivos	3
								Consultaron atención médica en medicina general	164	Consultaron atención médica en una clínica ambulatoria	23				
										Consultaron atención médica en emergencias	16				

Fuente: Elaboración propia con base en White *et al*.^1^; Green *et al*.^22^; Fukui *et al*.^23^; Fukui *et al*.^24^; Stewart *et al*.^25^; Hoffmann *et al*.^26^; Kim y Choi^27^; Lee *et al*.^28^; Yosef *et al*.^29^; y Giezendanner ^et al^.^30^. Notas: El guión (-) expresa que la categoría no fue incluida en el estudio. Las diferencias terminológicas reflejan los términos utilizados en los artículos originales, más allá de lo cual se intentó homogeneizar dichos términos para facilitar la lectura.

**Figura 2a:**
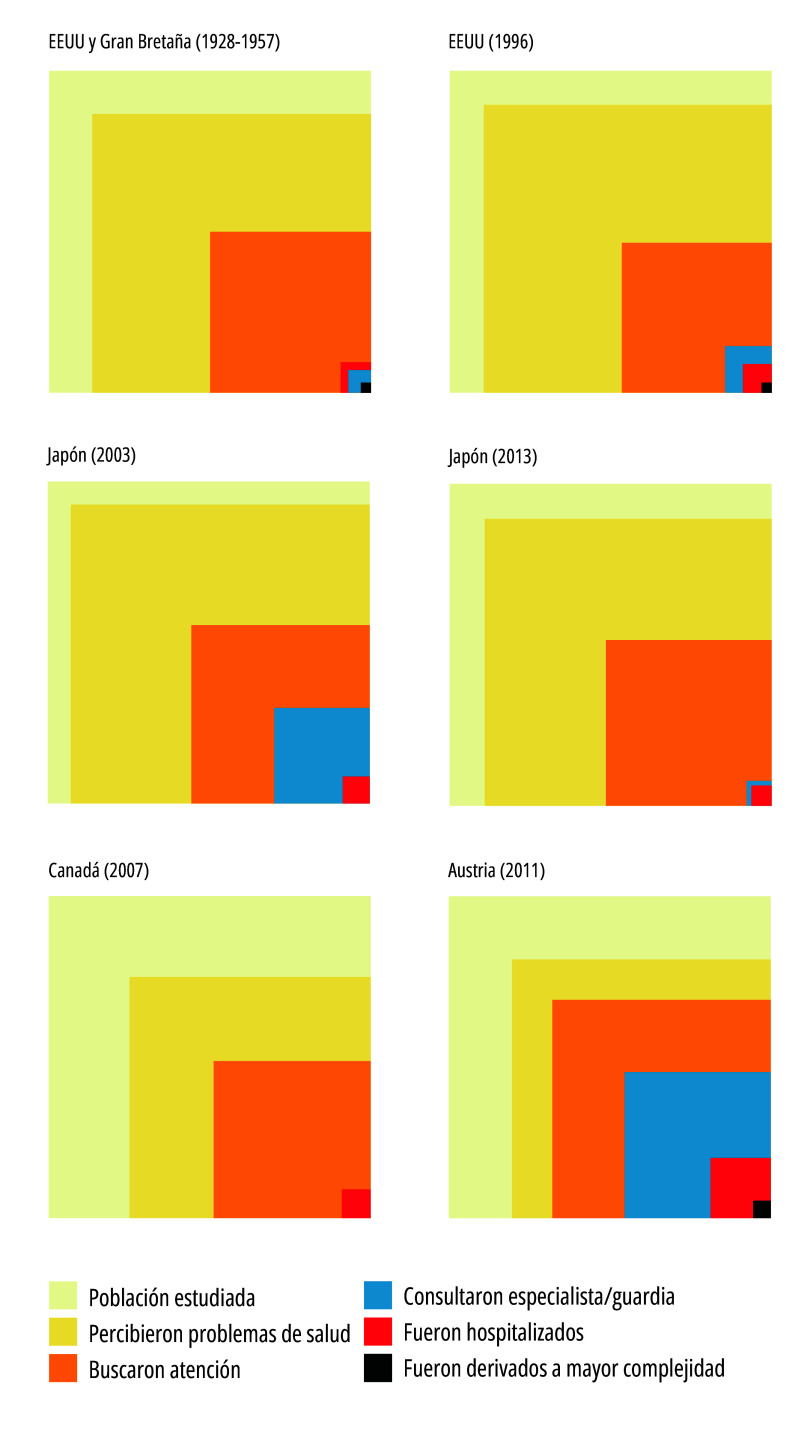
Resultados de los estudios de ecología de atención médica de EEUU y Gran Bretaña, EEUU, Japón, Canadá y Austria.

Fuente: Elaboración propia con base en White *et al*.^1^; Green *et al*.^22^; Fukui *et al*.^23^; Fukui *et al*.^24^; Stewart *et al*.^25^; Hoffmann *et al*.^26^ Nota: Los cuadrados de cada categoría agrupada de las figuras -categorías compatibles con las White *et al*. ^1^- representan el mayor valor obtenido para cada una de las diferentes categorías que integran las categorías agrupadas de los artículos analizados. Para estos fines, la categoría agrupada “Buscaron atención” se graficó en forma unificada.

**Figura 2b:**
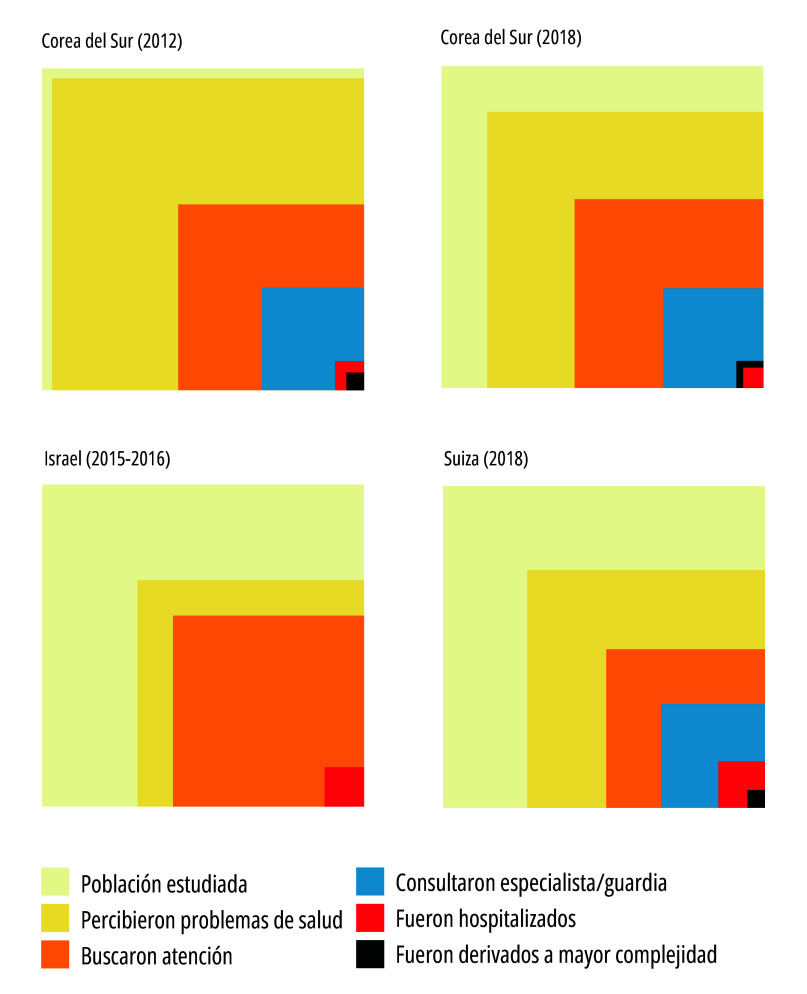
Resultados de los estudios de ecología de atención médica de Corea del Sur, Israel y Suiza.

Fuente: Elaboración propia con base en Kim y Choi^27^; Lee *et al*.^28^; Yosef *et al*.^29^; y Giezendanner *et al*.^30^. Nota: Los cuadrados de cada categoría agrupada de las figuras -categorías compatibles con las White *et al*. ^1^- representan el mayor valor obtenido para cada una de las diferentes categorías que integran las categorías agrupadas de los artículos analizados. Para estos fines, la categoría agrupada “Buscaron atención” se graficó en forma unificada.

A partir de los datos extraídos de los artículos se realizaron figuras siguiendo el modelo de White *et al*.^1^, respetando la proporción de población implicada en cada una de las categorías. Para facilitar la interpretación, estas figuras no dan cuenta de la subdivisión de la categoría “buscaron atención”, la cual puede observarse en la Tabla 1. Las figuras fueron realizadas mediante el software libre R, versión 4.2.1.

Los artículos fueron ordenados cronológicamente según el año de estudio, a excepción de los correspondientes al mismo país, de manera tal de permitir la comparación y sus equivalencias con los hallazgos del estudio de White *et al.*^1^. 

## RESULTADOS

La Figura 2a y la Figura 2b reúnen los resultados de los nueve estudios seleccionados, los cuales utilizaron el mismo estilo de gráfico adoptado por White *et al*.^1^. 

Green *et al*.^22^, con datos de 1996 de EEUU, publicaron una actualización del trabajo “*The ecology of medical care*” realizado 40 años atrás^1^. Utilizaron para ello una encuesta nacional de salud, representativa de la población de EEUU y una encuesta adicional representativa de la población nacional, a cuyos integrantes se contactó telefónicamente a fin de evaluar la proporción de personas que consideraron buscar atención médica y que consultaron a proveedores de medicina complementaria o alternativa, categorías que no utilizó el trabajo original. Los autores destacan la similitud entre sus resultados y los obtenidos en el trabajo original de White *et al.*^1^. 

En Japón, se realizaron dos estudios que replican la metodología para los años 2003 y 2013^23^^,^^24^. La muestra se realizó *ad-hoc* para los trabajos en cuestión y fue representativa de la población nacional de todas las edades, encargándose a un adulto las respuestas de los menores de 13 años. La encuesta se implementó en octubre de cada año para ambos estudios. Se registraron reportes diarios de síntomas, eventos relacionados con la salud y la decisión que se tomó. Los autores refieren que sus resultados fueron similares a los obtenidos en el estudio de White *et al.*^1^^)^ (Tabla 1). Al comparar los resultados de ambos trabajos se observó una disminución de la frecuencia de personas con síntomas, de las consultas a atención primaria y a consultorios externos hospitalarios, así como la utilización de medicaciones de venta libre. Por el contrario, hubo un marcado aumento de la utilización de medicina complementaria o alternativa (Tabla 1).

En el estudio de Austria^25^, realizado con datos del año 2011, se efectuaron encuestas telefónicas a personas de 16 años de edad o más. Luego de la obtención de una muestra representativa de la población, se constató que de cada 1.000 personas buscaron atención médica 460 y se internaron 35, cifras más elevadas que en el artículo de White *et al.*^1^^)^ (Tabla 1). Los autores adjudican esa diferencia al predominio de la oferta privada de servicios y prestaciones, características del sistema de salud austríaco. En los resultados del trabajo los autores comentan:

…La interpretación política de la libre elección de proveedores en Austria implica un acceso no regulado de los pacientes a todos los niveles de atención, incluso a través de la derivación, por ejemplo. Esto ha creado un sistema con una alta utilización en general, especialmente destacada en los sectores de atención secundaria y terciaria, con tasas de utilización cuatro veces mayores que las encontradas en Estados Unidos. El exceso de pacientes que acuden a hospitales universitarios para atención de rutina ha generado una carga en las estructuras de atención y el personal en las instituciones de atención terciaria, que deberían centrarse en brindar atención especializada para enfermedades raras y complejas.^25^

En Corea del Sur también se realizaron dos estudios que emplearon la misma metodología para los años 2012 y 2018^26^^,^^27^. En ambos casos, se utilizaron datos secundarios de encuestas nacionales de salud y encuestas adicionales con preguntas ampliadas a partir de muestras basadas en datos censales. La población de estudio incluyó a personas de 18 años o más, en el estudio de 2012, en tanto que para la encuesta de 2018 el punto de corte incluyó a los legalmente mayores de edad, es decir, a las personas de 19 años y más. Los resultados muestran que hubo más personas con problemas de salud y más hospitalizaciones que en el estudio de White *et al*.^1^. Los resultados de ambos estudios identificaron una disminución de la cantidad personas que reportaron problemas de salud entre 2012 y 2018. En contrapartida, hubo pocos cambios en la cantidad de personas que consultaron y atiendieron sus problemas de salud con médicos en clínicas y médicos en hospitales (Tabla 1). 

El estudio de Canadá de 2015^28^ utilizó un recorte poblacional diferente, que incluyó la población de 15 años o más, a partir de una encuesta telefónica con telefonía celular y fija. En este estudio se identificaron diferencias en la consulta médica por especialidad, observándose una mayor demanda a médicos generalistas por sobre otras especialidades (Tabla 1). Sin embargo, los resultados del número total de personas que consultaron y que fueron internadas fueron muy similares a los del estudio original de White *et al*.^1^.

El estudio realizado en Israel en dos momentos, en julio de 2015 y en agosto de 2016^29^ se llevó a cabo mediante una encuesta telefónica a personas de 15 años o más, que habitaban un hogar, de una muestra representativa de hogares del país. Debido a la baja representación de personas menores de 44 años o menos, se decidió complementar la muestra mediante encuestas realizadas por correo electrónico, lo cual, tal como aclaran los autores, torna a la muestra como no representativa de la población. En este contexto, los hallazgos del estudio en Israel mostraron que reportaron síntomas 495 personas por cada 1.000, sensiblemente más bajo que en el trabajo original de White *et al.*^1^*,* mientras que, por el contrario, la proporción de personas que consultaron al sistema de salud y que fueron internadas fue mayor. 

En Suiza, en el año 2018^30^, se realizaron encuestas a personas de 18 años o más a través de llamadas telefónicas que incluyeron telefonía celular y fija. La cantidad de personas que reportaron síntomas fue menor en comparación al estudio de White *et al.*^1^ Finalmente, las hospitalizaciones fueron elevadas en comparación al estudio de referencia (Tabla 1). Sin embargo, al igual que en los restantes estudios, el número total de hospitalizaciones fue bajo.

## DISCUSIÓN

En líneas generales, se observa una gran similitud entre los resultados de cada uno de los estudios de ecología de la atención médica a nivel país y los obtenidos en el artículo original de Kerr White *et al*.^1^, particularmente en lo que refiere al bajo número de hospitalizaciones y derivaciones a centros de mayor complejidad y, en aquellos países donde se repitió el estudio, los resultados fueron similares.

Entendemos que los estudios de la ecología de la atención médica van más allá de la cuestión biomédica, relacionando dimensiones poblacionales, biológicas, sociales y culturales. 

Los resultados de los trabajos analizados los discutiremos siguiendo tres dimensiones: Las regularidades en los resultados de los estudios de ecología de la atención médica; los sistemas de información en salud y las encuestas de salud; y el hospital y su hegemonía institucional en el campo de la salud. 

### Las regularidades en los resultados de los estudios de ecología de la atención médica

Más allá de las particularidades que se encuentran en los estudios de ecología de la atención médica analizados, que utilizan datos que abarcan 90 años de ocho países de distintos continentes y culturas, hay regularidades en los resultados relacionados con la gran proporción de personas que refieren problemas de salud y no consultan a la medicina científica y al bajo número de personas que son hospitalizadas o derivadas a centros de mayor complejidad. Esos resultados interpelan el sentido común y las narrativas científicas dominantes en el campo de la salud. Para analizar y discutir estos hallazgos nos vamos a centrar en dos preguntas: ¿cómo entender esas regularidades? y ¿qué hacer frente a ellas?

La respuesta a la primera pregunta la abordamos desde el concepto de regularidad planteado por Emile Durkheim^31^, y retomado por Pierre Bourdieu^32^^,^^33^^,^^34^^,^^35^^,^^36^^,^^37^^,^^38^ entre otros cientistas sociales. 

Durkheim señaló la uniformidad en la reproducción de los fenómenos sociales ante iguales circunstancias, y que esas uniformidades crean una ilusión de transparencia y de dominio inmediato del mundo social que las constituye: “…los hechos más arbitrarios en apariencia presentan después al observador atento, rasgos de constancia y de regularidad, síntomas de su objetividad”^31^. Y señala que para cambiarlos no basta la voluntad, sino que se requiere un gran esfuerzo y aun así pueden no cambiarse^31^. 

Para Bourdieu, las realidades sociales son construcciones históricas en las que la sintonía entre el campo y el *habitus* construyen un sentido práctico, concepto que entiende como una práctica sin conceptos que legitima el orden social^33^. Para Bourdieu, el campo es una configuración de relaciones entre posiciones objetivas, jerarquías, capitales y luchas en un espacio social estructurado por bienes materiales y simbólicos^33^^,^^37^^,^^39^, y el *habitus* un conjunto de disposiciones y esquemas de percepción incorporados que permiten construir el consenso que legitima el orden social a través de estrategias de reproducción y dominación social^37^^,^^38^. El *habitus* tiene un doble aspecto: reproduce los condicionamientos sociales, pero al mismo tiempo puede combinarlos en tanto “sistema de disposiciones inconscientes producido por la interiorización de estructuras objetivas”^33^. El *habitus* tiende a producir prácticas “objetivamente adherentes a las estructuras objetivas”^33^. El campo produce un dinamismo inscripto en las estructuras objetivas (objetividad de primer orden) y en las estructuras subjetivas (objetividad de segundo orden), estas últimas constituye el *habitus*^33^^,^^39^. 

Todo ello se expresa en las disposiciones de los agentes, entendidas como posiciones dinámicas que asumen en el campo^33^^,^^37^^,^^39^, que es el producto de una creación deliberada, que no está dada, sino que es una construcción histórica, que no sigue reglas, pero si tiene regularidades, no explícitas, ni codificadas^33^, y los jugadores juegan porque el juego merece ser jugado, no lo hacen por un contrato^33^^,^^39^. Y ese “merece ser jugado” es la expresión de modos de ver, sentir y actuar de los agentes, de percibir la realidad, que aunque parezca natural, está moldeada por las estructuras sociales^35^^,^^39^.

El producto de la conjugación entre campo y *habitus* genera regularidades difíciles de analizar dado que están integradas en la objetividad y en la subjetividad. Para poder trabajarlas es necesario objetivar tanto las estructuras objetivas, como las estructuras incorporadas bajo la forma de procesos mentales por medio de las cuales pensamos lo social, y que están ocultas por su eficacia en la experiencia social de los sujetos, los cuales las vivencian como evidentes^33^^,^^35^. Este trabajo pretende colaborar en ese proceso de objetivación. 

¿Qué hacer frente a los resultados que se obtienen de las investigaciones sobre ecología de la atención médica? Proponemos un abordaje desde el concepto de publicización, que plantea hacer públicos los problemas con el propósito de que sean parte de la agenda de la sociedad civil y la sociedad política^40^^,^^41^ por fuera de la influencia de la medicina y de otros intereses dominantes en el campo de la salud^42^. 

Distintos indicadores muestran que las regularidades en el campo de la salud no significan que todas las dimensiones mantengan la misma regularidad. El ejemplo más evidente es el gasto en salud, que tiene una tendencia constante al aumento, esa irregularidad se constituye en su propia regularidad. Un estudio de utilización de servicios y gastos en salud realizado en EEUU comparó los años 1996-1997 vs. 2011-2012^43^^)^ y reportó que, pese a que apenas se modificó el número promedio de personas que visitaron a un médico y la utilización total de servicios médicos entre esos dos períodos de tiempo, los gastos totales aumentaron notablemente (47,2% de aumento, pasando de u$246 dólares por persona al mes en 1996-1997 a u$362 dólares por persona al mes en 2011-2012, ajustado por inflación). Los aumentos en el gasto variaron drásticamente según la categoría, el aumento más notable se dio en medicamentos recetados, donde el gasto aumentó en un 159%, pasando de u$31 dólares por mes a u$80 dólares por mes. Las únicas dos categorías que no registraron un aumento fueron las visitas a atención primaria y las visitas domiciliarias, que se ubicaron respectivamente en el orden de los u$19 y de los u$14 dólares por mes de gasto en ambos períodos^43^. 

### Los sistemas de información en salud y las encuestas de salud

Kerr White tuvo una permanente preocupación por la salud de las personas, la efectividad y calidad de las prestaciones y los sistemas de información en salud^44^. Un ejemplo que expresa esa preocupación es la forma en que el mismo Kerr White entiende la figura de cuadrados anidados, al señalar que si nos ubicamos en el vértice superior izquierdo, veremos las dinámicas poblacionales constituidas por las trayectorias de las personas ante sus malestares físicos o emocionales, expresión de subjetividades y culturas; mientras que, si nos ubicamos desde el vértice inferior derecho tenemos una mirada desde los servicios de salud, y encontramos las categorías diagnósticas biomédicas tras las cuales tienden a desaparecer el sujeto, su historia y su contexto^44^.

En 2003, White criticó el modelo reduccionista biomédico que utilizan los sistemas de información en salud, porque terminan por cosificar las enfermedades bajo una concepción mecanicista de la condición humana^45^. Para White, la cosificación de las patologías oculta las necesidades de las personas que demandan atención-cuidado. Esa crítica le cabe tanto a la Clasificación Internacional de Enfermedades (CIE) de la Organización Mundial de la Salud como a la Systematized Nomenclature of Medicine (SNOMED) propuesta por el College of American Pathologists^45^ para ser utilizada en el primer nivel de atención. El SNOMED ha tomado gran difusión con la propuesta de las historias clínicas informatizadas, abriendo a su vez una gran puerta para negocios poco transparentes en su aplicación.

White cuestiona los objetivos de los sistemas de información en salud, y evidencia la poca jerarquía que los mismos tienen en la definición de las políticas y en la toma de decisiones por los gestores en la mayoría de los países. Considera, además, que habría que volver a la idea de George Engel del “laberinto de la atención médica”, que sostiene que los sistemas de información en salud debieran centrarse en la experiencia y el contexto de los pacientes para determinar el uso de cualquier nomenclatura^46^. White cita sus propias experiencias y de sus colegas como otras formas de registrar los datos de manera que el registro no cosifique al sujeto^47^^,^^48^. Lamentablemente, esas sugerencias han tenido poco éxito.

Entre 1964 y 1976, desde el departamento de *Health Services Research Center* de *Hopkins University* que él dirigía, y con el patrocinio de la Organización Mundial de la Salud (OMS) se lanzó el *Estudio colaborativo internacional sobre utilización de la atención médica* (OMS/ISMCU), que se propuso observar el uso de los servicios de salud en poblaciones definidas de diferentes áreas de siete países: Argentina, Canadá, EEUU, Finlandia, Gran Bretaña, Polonia y Yugoslavia^47^.

La experiencia en Argentina de ese estudio colaborativo se realizó en el marco del *Estudio sobre Salud y Educación Médica*, entre 1968-1973, a través de la Secretaría de Salud Pública de la Nación, quien llevó adelante un relevamiento de las condiciones sanitarias de cinco centros urbanos (Áreas Metropolitana, Córdoba, Mendoza, Rosario y Tucumán) y siete regiones (Pampeana, Centro, Cuyo, Comahue, Patagonia, Noroeste y Noreste). Con los datos relevados se produjeron estadísticas exhaustivas, sobre la utilización y el acceso de la población a los servicios de salud, la distribución y formación del personal de salud, el consumo de medicamentos y los recursos tecnológicos disponibles, entre otros. El fondo *Estudio sobre Salud y Educación Médica* se encuentra alojado en el Centro de Documentación Pensar en Salud del Instituto de Salud Colectiva de la Universidad Nacional de Lanús^49^ y reúne las publicaciones realizadas en el marco de ese estudio, en las cuales se plasmaron los principales resultados obtenidos en 26 informes realizados con tarjetas perforadas, procesadas por computadoras. Esos resultados hoy, en tiempos de inteligencia artificial, parecen imposibles de obtener.

Los resultados obtenidos en este trabajo muestran la relevancia de las encuestas de salud, que debieran ser un elemento central en los análisis de la situación de salud de una población, y no deben confundirse con las denominadas encuestas de factores de riesgo centrados en los estilos de vida, ni tampoco con las encuestas de utilización y gastos en salud^50^. Las encuestas de salud debieran realizarse de manera regular y no limitarse a las jurisdicciones nacionales, sino también abarcar escalas geográficas menores, como provincias y municipios, acción que inscribimos en la necesidad de avanzar en la implementación de la epidemiología de los servicios y sistemas de salud^42^ en diálogo con los conceptos de trayectoria del paciente, planteado desde las ciencias sociales^51^^,^^52^^,^^53^^,^^54^^,^^55^^,^^56^^,^^57^. Sostenemos, como muy necesario, instalar miradas múltiples sobre el campo de la salud para poder conocer y actuar sobre esos laberintos que se construyen entre las subjetividades, las culturas, las profesiones y los intereses del capital.

Los problemas de los sistemas de información en salud y la falta de desarrollo de la epidemiología en sistemas y servicios de salud señalados por White siguen teniendo vigencia^58^^,^^59^^,^^60^^,^^61^^,^^62^^,^^63^. La no resolución de esos problemas se explica desde el interés del desinterés de los principales agentes del campo de la salud^42^.

### El hospital y su hegemonía institucional en el campo de la salud

La gran reforma médica que se produjo en EEUU a partir del *Informe Flexner*^64^, dio inicio a un proceso de consolidación de la medicina científica, que se expandió en las décadas siguientes a nivel mundial e institucionalizó al hospital como centralidad de los procesos de atención de las personas^6^^,^^64^^,^^65^. 

El resultado de una institucionalidad centrada en el hospital contribuyó a la deshumanización de la atención y a la pérdida progresiva de la motivación en el trabajo de un número cada vez más importante de profesionales, que se agravó a partir de la pandemia de covid-19. Esa elección institucional favoreció y favorece la expoliación de los presupuestos públicos, y de los bolsillos de las personas por los pagos directos e indirectos para acceder a consultas o tratamientos, lo cual se agravó con la consolidación del complejo médico industrial a partir de la década de 1970^6^^,^^64^^,^^66^^,^^67^^,^^68^. 

Los estudios de ecología de la atención médica demuestran las limitaciones de la figura del hospital como institucionalidad hegemónica para el proceso de atención y cuidado de las personas y la necesidad de construir otra hegemonía institucional al interior del campo de la salud, basada en centros de salud vinculados a lo territorial, y pensados como instituciones a escala humana y no a escala fabril, como devino la institución hospitalaria^66^.

## CONCLUSIONES

Los estudios de ecología de la atención médica analizados abarcan 90 años en diferentes países y continentes, durante los cuales se produjeron grandes transformaciones en los métodos de diagnóstico y tratamiento, con la incorporación de nuevos medicamentos y tecnologías que no siempre fueron necesarios^70^^,^^71^. A pesar de todo ello, las regularidades encontradas en los resultados son llamativas y debieran ser insumos en las discusiones que se realizan sobre la atención de las personas y el gasto en salud, procurando responder ¿dónde y en qué invertir?; ¿qué institucionalidad se necesita para atender las demandas del proceso salud-enfermedad-atención-cuidado en las poblaciones?; ¿qué relevancia tienen, para los conjuntos sociales, los procesos de autoatención y el acceso a otras racionalidades médicas, no incluidas en la medicina científica, como la homeopatía, la medicina ayurvédica, la acupuntura, y la medicina de los pueblos originarios?

White critica la naturalización que se produjo en la separación entre clínica, epidemiología y salud pública^65^, separación totalmente naturalizada en los hospitales y ministerios, pero artificial en los territorios donde los problemas no se estructuran siguiendo la fragmentación de los saberes sino que se expresan por los problemas de las personas y los conjuntos poblacionales^69^.

Por todo lo anterior, resaltamos la importancia de los estudios de ecología de la atención médica y la publicización de sus resultados de manera que se puedan transformar en problema público^40^; ya que, como bien señaló Mario Testa, los cambios en el campo de salud no vendrán desde el propio campo sino de las fuerzas organizadas por fuera del campo de la salud que realicen demandas al Estado y construyen ciudadanía social^72^.
