# Supplementary material for: Estudios de ecología de la atención médica: análisis comparado en dimensión histórica, 1928-2018
Source: Salud Colect. 2023 Nov 22;19:e4549. [Article in Spanish] doi: 10.18294/sc.2023.4549 (PMC11930360; doi:10.18294/sc.2023.4549)
Supplement: Translation

# Translation: Studies on the ecology of medical care: a comparative analysis in historical perspective, 1928-2018

**Authors:** Hugo Spinelli, Andrés Trotta, Viviana Martinovich, Marcio Alazraqui

**Affiliations:** 1 Doctor in Collective Health. Researcher, Instituto de Salud Colectiva, Universidad Nacional de Lanús, Buenos Aires, Argentina. hugospinelli09@gmail.com; 2 Doctor in Collective Health. Director, Master’s Degree in Epidemiology, Management and Health Policies, Instituto de Salud Colectiva, Universidad Nacional de Lanús, Buenos Aires, Argentina. doctortrotta@gmail.com; 3 Assistant Professor, Instituto de Salud Colectiva, Universidad Nacional de Lanús, Buenos Aires, Argentina. vivianamartinovich@gmail.com; 4 Doctor in Collective Health. Director, Instituto de Salud Colectiva, Universidad Nacional de Lanús, Buenos Aires, Argentina. malazraqui@yahoo.com

**Keywords:** Medical Care, Information Systems, Surveys, Hospitals

## Abstract

In the definition of health policies and decision-making on the part of health officials, there is often a prevailing separation between clinical practice, epidemiology, and public health. Although this division is naturalized from the viewpoint of hospitals and public agencies, it is artificial in the context of concrete territories and communities, where problems are not structured according to the fragmentation of knowledge, but rather express the complexities of the problems faced by individuals and population groups. In this context, this article compiles and analyzes studies on the ecology of medical care carried out between 1928 and 2018 that have revisited the pioneering study “The ecology of medical care” by White, Williams and Greenberg. The discussion is structured around three central issues: 1) recurrent themes in studies on the ecology of medical care; 2) health information systems and health surveys; and 3) the institutional hegemony of hospitals in the health field.

## INTRODUCTION

In 1961, the *New England Journal of Medicine* published the article “The ecology of medical care”. In that article, Kerr White, Franklin Williams, and Bernard Greenberg^1^ posed three questions: is the burgeoning harvest of new knowledge fostered by immense public investment in medical research being delivered effectively to the consumers?; Is the available quantity, quality, and distribution of medical care optimum in the opinion of the consumers?; and, whose responsibility is it to examine these questions and provide data upon which sound judgments and effective programs can be based? These questions are still relevant today in every country. The authors^1^ state that little is known about the reasons why people seek help when they perceive some disturbance in their sense of well-being and where they seek it. Furthermore, they acknowledge that the acceptance and use of medical care are processes under the control of individuals themselves.

In that paper, they analyzed the decisions made by individuals aged 16 and older in response to illnesses or injuries affecting their well-being. They used six categories: adult population exposed to risk, adults reporting one or more illnesses or injuries per month, adults consulting a physician one or more times per month, adult patients admitted to a hospital per month, adult patients referred to another physician per month, and adult patients referred to a university medical center per month^1^. The first two categories pertain to the population, the third to seeking medical attention, and the last three to medical care^1^. The final results revealed that out of every 1,000 people, 750 reported one or more illnesses or injuries, 250 consulted a doctor one or more times, 5 individuals were referred to another doctor, 9 individuals were hospitalized, and only 1 was referred to a university medical center^1^.

Some noteworthy methodological details include that the time unit was the month, the unit of analysis was the individual’s response to their illnesses or injuries, and the decisions of physicians, rather than focusing on the diagnosis of the disease. This approach goes against the prevailing culture in health information systems, in which events are usually recorded based on disease diagnoses. The study also excluded uncomplicated pregnancies and individuals under the age of 16, since the authors consider that decisions in these cases were often made by their parents^1^.

The work by White et al.^1^ was based on publications from the 1950s and the early 1960s^2^^,^^3^^,^^4^^,^^5^. They used data sources from two different countries: US (reports of the Committee on the Costs of Medical Care, based on a country representative sample of the white population between 1928 and 1931), and England and Wales (Survey of Sickness, based on a country representative sample of the population of between 1946 and 1950).

**Figure 1:** Estimates of the monthly prevalence of disease in the community and the roles of physicians, hospitals, and university medical centers in the provision of medical services (adults 16 years and older). USA and Great Britain (1928-1957).

Source: Own elaboration based on White *et al*.^1^

In their study, White *et al*.^1^ pointed out that only in a small proportion of the times they suffer discomforts, illnesses and symptoms. They graphed their findings through a nesting square diagram (Figure 1), based on Horder and Horder^2^, who had previously described the pattern of diseases that a general practitioner in London received back in 1954 with that diagram. Horder and Horder studied 2,000 medical consultations during the summer and winter quarters, providing insights into the profile of people’s illnesses within the general practitioner visits. The unit of analysis they used was the first medical consultation, rather than the disease itself. The innovative aspect of this case study was its focus on recording the relative frequency of specific medical conditions during the first consultations, as opposed to measuring the relative frequency of a particular disease within all consultations, which the most common method. This work strongly influenced the research of White *et al*.^1^.

The historical period during which the paper by White et al. was published corresponds to what Starr refers to as the liberal years of healthcare in the United States. This era was characterized by the rise of a hospital-centered culture, as reflected in the actions of the U.S. Congress, which created the federal program known as Hill-Burton. This program aimed to increase by 40% the total number of hospital beds in the country by creating 140,000 new hospital beds^6^^,^^7^^,^^8^.

In 1996, 35 years after the publication of his article, White mentioned that he received numerous angry letters from colleagues due to the article’s content at the time of its publication. This led him to believe that the work would be quickly forgotten^9^. He also stated that he repeated the analysis of the 1961 publication^1^ using data from the 1973 National Center for Health Statistics, and the results remained consistent. He also emphasized the importance of the general practitioner and regretted that he was unaware of the 1920 Dawson Report from England when he wrote the 1961 paper. The Dawson report introduced the concept of primary medical care and proposed that the core healthcare institutions should be primary health centers, located within the communities, with hospitals serving as secondary health centers for reference^10^.

White also noted the resistance he encountered from the editor of the New England Journal of Medicine, Joe Garland, when trying to include the word “ecology” in the article’s title. Although he did not explain the reason for using the term “ecology,” there are references that can help us understand some of the influences they may have based on.

The term “ecology” was first published as the German word “*Ökologie*,” coined in 1866 by the zoologist and biologist Ernst H. Haeckel (1834-1919). It is derived from the Greek words “*oîkos*” (house) and “lógos” (study), referring to the study of the environment in which living organisms develop. However, the concept was not widely used until about two and a half decades after its formulation, when Ellen Swallow Richards (1842-1911) began using it in the United States.

Ellen Swallow Richards holds a significant place in history as the first woman to attend a science university and the Massachusetts Institute of Technology (MIT) in the United States. She played a pioneering role in the intersection of chemistry and nutrition^11^^,^^12^. Richards is considered one of the early feminists in the United States, who led a revolution of domestic eating practices^13^. In 1870, she and Mary Hinman Abel established a school to train impoverished women in the proper feeding of their families^11^. She is regarded as one of the founders of nutrition, consumer economics, environmental hygiene, and ecological science^14^^,^^15^^,^^16^. The *Journal of The American Public Health Association* published her obituary in 1911, underscoring her significance in the field of public health^17^.

Another reference in the context of ecology comes from Robert Ezra Park (1864-1944), one of the early figures of the Chicago School of Sociology, who was influenced by John Dewey at Harvard University. Park is considered one of the leading figures of this school, along with Thomas William^18^. Park advocated the idea that physical space mirrors social space, leading him to formulate the notion of ecology, not in the contemporary sense but borrowing from biology, where it refers to the competition of plant and animal for space^19^^,^^20^^,^^21^. It is conceivable that White adopted this ecological concept for his work.

The study by White *et al*.^1^ was replicated in several countries decades later. In the present study, we aim to conduct a comparative analysis between the results of White *et al*.^1^ and the ecology of medical care studies which addressed countries as a whole. The discussion will focus on three main issues: 1) regularities found in the ecology of medical care studies; 2) health information systems and health surveys; and 3) the institutional hegemony of hospitals in the field of healthcare.

## METHODS

A bibliographic search of scientific articles following the model used by White et al.^1^ in “The ecology of medical care” was conducted to assemble a bibliographic corpus of available literature. The search was performed in the following databases: Scopus, PubMed, SciELO, and Virtual Health Library. The combination of terms and logical operators used in each of the four mentioned bibliographic databases was: a) “ecology of medical care” and “health services”; b) “ecology of medical care” and “health services research”; y c) “ecology of medical care” and “health services utilization”. 

The bibliographic search was conducted in July 2023, encompassing articles that underwent analysis at the country level and selecting those that employed the graphical representation of the nested squares model by White et al.^1^ Articles with results at a lower level of aggregation than the country - such as province, municipality, or equivalent - or in situations related to territorial disputes like Taiwan and Hong Kong were not included.

The corpus consisted of nine articles from eight countries^22^^,^^23^^,^^24^^,^^25^^,^^26^^,^^27^^,^^28^^,^^29^^,^^30^. Two articles were found for both Japan and South Korea. For the analysis of the selected articles, the results were grouped into categories compatible with those used by White et al. in 1961^1^: a) Studied population; b) perceived health problems; c) sought medical attention; d) consulted a specialist/emergency department; e) were hospitalized; and f) were referred to higher complexity. The analysis did not include the category “referred to another physician,” as used by White et al., since it was not replicated in any of the other studies. In addition, for the category “sought medical attention,” a subdivision into two subcategories was established: “sought medical attention: biomedical practices” and “sought medical attention: alternative practices to biomedicine,” as four of the analyzed articles used this division^22^^,^^23^^,^^26^^,^^28^, which was considered relevant (Table 1).

**Table 1:** Number of people in the categories adapted from Kerr White’s proposal, according to countries and periods included in this work.

Country	Study period	Study population	n	Perceived health problems	n	Sought medical attention				Consulted specialist/guard	n	Were hospitalized	n	Were referred to higher complexity	n
						Alternative practices to biomedicine	n	Biomedical practices	n						
USA and Great Britain^1^	1928-1957	Population aged ≥16	1,000	Adults reporting one or more illnesses or injures per month	750	-	-	Adults consulting a physician one or more times per month	250	Adults patient referred to another physician per month	5	Adults patients admitted to a hospital per month	9	Adult patient referred to a university medical center per month	1
USA^22^	1996	General Population	1,000	Report symptoms	800	Complementary or alternative medical care provider	65	Visit a physician’s office	217	Hospital outpatient clinic	21	Hospitalized	8	Hospitalized in an academic medical center	1
				Consider seeking medical care	327			Visit a primary care physician’s office	113						
								Home health care	14	Emergency department	13				
Japan^23^	2003	General population	1,000	Report symptoms	862	Visit a complementary or alternative medical care provider	49	Visit physician's office	307	Visit a hospital outpatient clinic	88	Are hospitalized	7	-	-
								Visit a primary care physician	232	Visit a university hospital outpatient clinic	6				
								Receive home health care	3	Visit an emergency department	10				
Japan^24^	2013	General population	1,000	Report symptoms	794	-	-	Visit physician office	265	Visit university medical center	10	Have hospitalization	6	-	-
								Visit primary care physician	206						
								Receive home health care	7	Visit emergency room						4
Canada^25^	2007	Population aged ≥15	1,000	Have 1 or more chronic conditions	561	-	-	Contact family physicians	238	-	-	Stay overnight in hospital	8	-	-
								Contact physicians other than family physicians	50						
								Contact nurses	32						
Austria^26^	2011	Population aged ≥16	1,000	Report symptoms	646	-	-	Seek any medical care	460	Visit a specialist in an ambulatory care setting	206	Are hospitalized	35	Are hospitalized in an academic medical center	3
				Consider medical care	530			Visit a general practitioner's office	336	Visit a specialist in a hospital outpatient facility	78				
South Korea^27^	2012	Population aged ≥18	1,000	Have a health problem	939	Visit an Oriental medical provider	38	Visit a clinic	333	Visit a hospital outpatient department	101	Are hospitalized in a clinic	3	Are hospitalized in a tertiary hospital	3
										Visit a tertiary hospital outpatient department	35	Are hospitalized in a hospital	8		
										Visit an emergency department	7				
South Korea^28^	2018	Population aged ≥18	1,000	Have a health problem	763	-	-	Visit a clinic	344	Visit a secondary hospital outpatient department	56	Are hospitalized in a secondary hospital	4	Are hospitalized in a tertiary hospital	7
										Visit a tertiary hospital outpatient department	96				
										Visit an emergency department	9				
Israel^29^	2015-2016	Population aged ≥15	1,000	Report symptoms	495	-	-	Get medical assistance	352	-	-	Hospitalized	15	-	-
				Consider seeking medical care	450										
Switzerland^30^	2018	Population aged ≥18	1,000	Had symptoms	546	Contacted alternative medical care provider	7	Ask for medical advice	243	Visited a specialist	81	Had in-patient care	21	Required Intensive Care Unit	3
								Visited their general practitioner	164	Contacted an outpatient clinic	23				
										Self-admitted to Accident & Emergency	16				

Source: Own elaboration based on White *et al*.^1^; Green *et al*.^22^; Fukui *et al*.^23^; Fukui *et al*.^24^; Stewart *et al*.^25^; Hoffmann *et al*.^26^; Kim y Choi^27^; Lee *et al*.^28^; Yosef *et al*.^29^; and Giezendanner *et al*.^30^. Notes: The hyphen (-) indicates that the category was not included in the study. The terminological differences reflect the terms used in the original articles, beyond which an attempt was made to homogenize these terms to facilitate reading.

**Figure 2a:** Results of health care ecology studies from the USA and Great Britain, USA, Japan, Canada and Austria.

Source: Own elaboration based on White *et al*.^1^; Green *et al*.^22^; Fukui *et al*.^23^; Fukui *et al*.^24^; Stewart *et al*.^25^; Hoffmann *et al*.^26^ Note: The squares of each grouped category in the figures, categories compatible with White et al.^1^ represent the highest value obtained for each of the different categories that make up the grouped categories of the articles analyzed. For these purposes, the grouped category “Sought medical attention” was plotted in a unified form.

**Figure 2b:** Results of health care ecology studies from South Korea, Israel, and Switzerland.

Source: Own elaboration based on Kim & Choi^27^; Lee *et al*.^28^; Yosef *et al*.^29^; and Giezendanner *et al*.^30^. Note: The squares of each grouped category in the figures, categories compatible with White *et al*.^1^ represent the highest value obtained for each of the different categories that make up the grouped categories of the articles analyzed. For these purposes, the grouped category “sought medical attention” was plotted in a unified form.

Figures were created based on the data extracted from the articles, following the model of White et al.^1^, taking into account the proportion of the population for each category. To facilitate interpretation, these figures do not account for the subdivision of the “sought medical attention” category, which can be observed in Table 1. The figures were created using the free software R, version 4.2.1.

The articles were ordered chronologically by the studied period, with the exception of those from the same country, to allow for comparison and their equivalence with the findings of the study by White et al.^1^.

## RESULTS

The results of the nine selected studies can be seen in Figures 2a and 2b, which used the same style of graph adopted by White et al.^1^.

Using data from 1996 in the United States, Green et al.^22^ published an update of the work “The ecology of medical care” conducted 40 years earlier^1^. For this purpose, they used a national health survey representative of the U.S. population and an additional survey representative of the national population. The latter was contacted by phone to assess two categories not used in the original work: the proportion of people who considered seeking medical care and the proportion of people who consulted providers of complementary or alternative medicine. The authors highlight the similarity between their results and those obtained in the original study by White et al.^1^.

In Japan, two studies replicated the methodology for the years 2003 and 2013^23^^,^^24^. The sample was created specifically for the respective studies and represented the national population of all age groups. Adults were responsible for answering on behalf of children under 13 years old. The survey was conducted in October of each year for both studies. The data was recorded in daily reports of symptoms, health-related events, and the decisions made. The authors reported that their results were similar to those in White et al.^1^ (Table 1). When comparing the results of both Japan studies, a decrease in the frequency of people with symptoms, primary care consultations, outpatient hospital visits, and the use of over-the-counter medications was observed. In contrast, there was a significant increase in the use of complementary or alternative medicine (Table 1).

In the Austria study^25^ for the year 2011, telephone surveys were conducted within a representative sample of individuals aged 16 and over. The results for two categories were higher than in White et al.^1^: out of 1,000 people, 460 sought medical attention and 35 were hospitalized (Table 1). The authors attributed this difference to the predominance of private healthcare services, a characteristic of the Austrian healthcare system. In the results of the study, the authors commented:

“The political interpretation of free provider choice in Austria implies unregulated patient access to all levels of care including for example through self-referral. This has created a system with overall high utilization, especially prominent in the secondary and tertiary care sectors with utilization rates four times those found in the USA (see figure 1). Excessive numbers of patients attending university hospitals for routine care have created a burden on care structures and staff in tertiary care institutions which should focus to deliver sophisticated care for rare and complex diseases.”^25^

In South Korea, two studies were also conducted using the same methodology for the years 2012 and 2018^26^^,^^27^. In both cases, secondary data from national health surveys and additional surveys with expanded questions based on census data samples were used. The study population included individuals aged 18 and older in the 2012 study, while the 2018 it included individuals aged 19 and older. The results show that there were more people with health problems and more hospitalizations than in White et al. study^1^. The comparison of both Korean studies show a decrease in the number of people reporting health problems between 2012 and 2018. Conversely, there were few changes in the number of people who consulted and addressed their health problems with physicians in clinics and hospital doctors (Table 1).

The 2015 study in Canada^28^ used a different population cutoff, including individuals aged 15 and older, from a telephone survey using both cell and landlines. This study identified differences in medical consultation by specialty, with a higher demand for general practitioners compared to other specialties (Table 1). However, the total number of people who consulted and were hospitalized was very similar to White et al. original study^1^.

The study conducted in Israel at two different times, in July 2015 and August 2016^29^, was carried out through telephone surveys of individuals aged 15 and older obtained from a representative sample of households in the country. Authors decided to supplement the sample with surveys conducted via email due to the low representation of individuals under 44 years of age or younger. As the authors clarify, this rendered the sample as non-representative of the population. In this context, the findings of the study showed that 495 people per 1,000 reported symptoms, significantly lower than in White et al. original work. Conversely, the proportion of people who consulted the healthcare system and were hospitalized was higher.

In Switzerland, surveys were conducted in the year 2018^30^ within individuals aged 18 and older through telephone calls, including both cell and landlines. The number of people reporting symptoms was lower compared to White et al. Hospitalizations, on the other hand, were higher (Table 1). However, as in the other studies, the total number of hospitalizations was low.

## DISCUSSION

A significant similarity is observed among the results of each of the country-level ecology of medical care study and those of White et al. original paper^1^, particularly regarding the low number of hospitalizations and referrals to higher complexity medical centers. In countries where the study was replicated in different years, the results also were similar to white et al findings. 

We understand that studies on the ecology of medical care extend beyond biomedical issues, linking population, biological, social, and cultural dimensions.

We will discuss the results of the analyzed studies based on three dimensions: the regularities found in the ecology of medical care studies, health information systems and health surveys, and the institutional hegemony of hospitals in the field of healthcare.

### Regularities found in the ecology of medical care studies

Beyond the specific findings in the analyzed studies, which span 90 years and cover eight countries from different continents and cultures, there are certain consistent patterns in the results. These patterns are related to the significant proportion of individuals who report health problems but do not seek medical attention from scientific medicine, as well as the low number of people who are hospitalized or referred to more specialized healthcare facilities. These results challenge common sense and prevailing scientific narratives in the field of healthcare. To analyze and discuss these findings, we will focus on two questions: how can we understand these regularities?, and what actions should be taken in response?

To address the first question, we refer to the concept of regularities introduced by Emile Durkheim^31^ and further developed by Pierre Bourdieu^32^^,^^33^^,^^34^^,^^35^^,^^36^^,^^37^^,^^38^ among other social scientists. Durkheim emphasized the uniformity in the reproduction of social phenomena under similar circumstances. He noted that these uniformities create an illusion of transparency and immediate mastery of the social world: “The most arbitrary facts, in appearance, present to the attentive observer signs of constancy and regularity, symptoms of their objectivity”^31^. Durkheim also pointed out that changing these regularities requires significant effort which may still be unsuccessful^31^.

According to Bourdieu, social realities are historical constructions in which the harmony between the field and habitus shapes a practical sense, a concept that he understands as a practice without explicit concepts that legitimizes the social order^33^. Bourdieu states that the field is a configuration of relationships between objective positions, hierarchies, capitals, and struggles within a social space structured by material and symbolic goods^33^^,^^37^^,^^39^. The habitus consists of a set of dispositions and incorporated perceptual schemes that allow the construction of consensus that legitimizes the social order through strategies of social reproduction and domination^37^^,^^38^. The habitus has a dual aspect: it reproduces social conditioning while also combining them as a “system of unconscious dispositions produced by the internalization of objective structures”^33^. The habitus tends to produce practices that are “objectively adherent to objective structures”^33^. The field generates a dynamism that is inscribed within both objective structures (first-order objectivity) and subjective structures (second-order objectivity), with the latter constituting the habitus^33^^,^^39^.

All of this is manifested in the dispositions of the agents, understood as dynamic positions assumed within the field^33^^,^^37^^,^^39^. The field is the result of a deliberate creation, it is not given but rather a historical construction. It does not follow explicit or codified rules, but it exhibits implicit and uncoded regularities^33^. Players engage in the field because the game is worth playing, not because of a formal contract^33^^,^^39^. This “worth playing” reflects the ways in which agents perceive, experience, and act upon reality, which is shaped by social structures, although it may appear natural^35^^,^^39^.

The product of the interaction between the field and habitus generates regularities that are challenging to analyze because they are integrated into both objectivity and subjectivity. To work with these regularities, it is necessary to objectify both objective structures and structures incorporated in the form of mental processes through which we understand the social world. These structures remain hidden due to their efficacy in the social experience of individuals, who perceive them as self-evident^33^^,^^35^. This work aims to contribute to the process of objectification.

As for the question of what to do in response to the results obtained from research in ecology of medical care, we propose an approach based on the concept of “publicization.” This approach involves making problems become public, with the purpose of including them on the agenda of civil society and political society, independent of the influence of the medical establishment and other dominant interests within the healthcare field^40^^,^^41^.

Various indicators demonstrate that the regularities in the field of healthcare do not mean that all dimensions remain the same. A prime example is healthcare expenditure, which consistently shows an upward trend, constituting its own kind of regularity. A study on healthcare service utilization and spending conducted in the United States compared the years 1996-1997 to 2011-2012^43^. It reported minimal changes in the average number of people visiting a physician and the overall utilization of medical services between these two time periods. However, the total healthcare spending increased significantly by 47.2%, rising from $246 per person per month in 1996-1997 to $362 per person per month in 2011-2012 (adjusted for inflation). The increases in spending varied dramatically across categories, with the most notable increase seen in prescription drugs, where spending rose by 159%, from $31 per month to $80 per month. The only two categories that did not show an increase in spending were consultations to primary care and home visits, which remained at approximately $19 and $14 per person per month, respectively, in both time periods^43^.

### Health Information Systems and Health Surveys

Kerr White maintained a consistent concern for people’s health, the effectiveness and quality of healthcare services, and health information systems^44^. One example that reflects his concern is the way he perceives the concept of the nested squares diagram. He noted that if we position ourselves at the upper left vertex, we can see the population dynamics constituted by people’s paths when dealing with their physical or emotional discomforts, which express subjectivities and cultures. On the other hand, if we position ourselves in the lower right vertex, we have a healthcare services perspective, and we encounter biomedical diagnostic categories that tend to hide the subjects, their history, and their context^44^.

In 2003, White criticized the reductionist biomedical model used by health information systems because it ends up in a reification of diseases under a mechanistic conception of human conditions^45^. White believed that the reification of pathologies obscures the needs of individuals seeking care. This criticism applies to both the International Classification of Diseases (ICD) by the World Health Organization and the Systematized Nomenclature of Medicine (SNOMED) proposed by the College of American Pathologists for use at the primary care level^45^. SNOMED has gained significant traction with the proposal of computerized medical records, which has also opened doors for opaque business practices in its application.

White questioned the goals of health information systems and highlighted their lack of influence in shaping policies and decision-making by healthcare decision makers in most countries. He argued that it would be wise to return to George Engel’s concept of the “healthcare maze,” which suggests that health information systems should focus on patients’ experiences and contexts to determine the use of any nomenclature^46^. White cited his own experiences with some colleagues as alternative ways to record data without objectifying the subject^47^^,^^48^. Unfortunately, these suggestions had a limited success.

Between 1964 and 1976, under White’s direction at the Center for Health Services and Outcomes Research at Johns Hopkins University, and with the sponsorship of the World Health Organization (WHO), the International Collaborative Study of Medical Care Utilization (WHO/ISMCU) was initiated. The study aimed to address healthcare service utilization in defined populations in different areas of seven countries: Argentina, Canada, the United States, Finland, the United Kingdom, Poland, and Yugoslavia^47^.

In Argentina, this collaborative study was conducted under the framework of the Health and Medical Education Study between 1968 and 1973, led by the National Secretary of Public Health. It involved a survey of the health conditions in five urban centers (Metropolitan Area of Buenos Aires, Córdoba, Mendoza, Rosario, and Tucumán) and seven regions (Pampa, Central, Cuyo, Comahue, Patagonia, Northwest, and Northeast). The study compiled comprehensive statistics about the utilization and access of the population to healthcare services, the distribution and training of healthcare personnel, medication consumption, and available technological resources, among other dimensions. The Health and Medical Education Study archive is located in the Thinking in Health Documentation Center of the National University of Lanús^49^. It contains publications that document the main results in 26 reports generated using punched cards and processed by computers. These results, especially in the age of artificial intelligence, seem nearly impossible to obtain nowadays.

The results from our study underscore the importance of health surveys, which should be a central element in analyzing a population’s health status. These should not be thought as surveys of risk factors centered on lifestyle, nor as surveys of healthcare utilization and spending^50^. Health surveys should be conducted regularly and extend beyond national jurisdictions to encompass smaller geographic scales, such as provinces and municipalities. This aligns with the need to advance the implementation of the epidemiology of healthcare systems and services^42^ in conjunction with the concepts of patient pathways as proposed by social sciences^51^^,^^52^^,^^53^^,^^54^^,^^55^^,^^56^^,^^57^. It is crucial to introduce multiple perspectives into the healthcare field to understand and address the mazes that emerge from subjectivities, cultures, professions, and capital interests.

The problems with health information systems and the lack of development in healthcare system and services epidemiology remain relevant, as pointed out by White^58^^,^^59^^,^^60^^,^^61^^,^^62^^,^^63^. The persistence of these issues can be attributed to the disinterest of key stakeholders in the healthcare field^42^.

### Institutional hegemony of hospitals in the field of healthcare

The significant medical reform that was held out in the United States following the Flexner Report^64^ initiated the process of consolidating scientific medicine, which expanded globally in the subsequent decades and institutionalized the hospital as the focal point of healthcare processes for individuals^6^^,^^64^^,^^65^.

The outcome of the institutional focus on hospitals has contributed to the dehumanization of healthcare and the gradual decline in motivation among an increasing number of professionals, a situation that worsened with the onset of the COVID-19 pandemic. This institutional choice favored and continues to favor the depletion of public budgets and individuals’ wallets through direct and indirect payments for consultations or treatments. This problem was exacerbated with the consolidation of the medical-industrial complex starting in the 1970s^6^^,^^64^^,^^66^^,^^67^^,^^68^.

Studies on the ecology of medical care reveal the limitations of the hospital as the hegemonic institution for the healthcare process and emphasize the need to construct an alternative institutional hegemony within the healthcare field. This alternative should be based on community health centers linked to geographical regions, designed as institutions on a human scale rather than as factory-like institutions, as hospitals have become^66^.

## CONCLUSIONS

The studies on the ecology of medical care that have been analyzed cover 90 years, different countries, and continents, during which significant transformations occurred in diagnostic and treatment methods, incorporating new drugs and technologies, not always necessary^70^^,^^71^. Despite these changes, the regularities found in the results are striking and should serve as a landmark for discussions about people’s healthcare and healthcare spending. These discussions should address questions such as: Where and what to invest in? What kind of institutions are needed to address the demands of the health-disease-care process in populations? What relevance for social groups are held by self-care processes and other medical rationalities, not included in scientific medicine, like homeopathy, Ayurvedic medicine, acupuncture, and indigenous medical practices?

White criticizes the naturalization of the separation between clinical practice, epidemiology, and public health^65^, a separation that is entirely naturalized in hospitals and ministries but artificial in the territories, where problems are not in line with the fragmentation of knowledge but are expressed through the issues faced by individuals and populations^69^.

For all the aforementioned reasons, we emphasize the importance of studies on the ecology of medical care and the public dissemination of their results, so that they can become public issues^40^. As Mario Testa rightly noted, changes in the healthcare field will not come from within the field itself but from organized actors outside the healthcare field that make demands to the State.
